# Wound Healing on the Cutting Plane of Ankle Bones after Incomplete Revascularization for Chronic Limb-Threatening Ischemia in an Elderly Patient: A Case Report

**DOI:** 10.3400/avd.cr.22-00049

**Published:** 2022-09-25

**Authors:** Shinsuke Kikuchi, Daiki Uchida, Kazuki Takahashi, Yuri Yoshida, Ai Tochikubo-Suzuki, Tomoki Nakatsu, Mineko Higuchi, Nobuyoshi Azuma, Kazuya Kato

**Affiliations:** 1 Department of Surgery, Pippu Clinic, Pippu-cho, Kamigawa-gun, Hokkaido, Japan; 2 Department of Vascular Surgery, Asahikawa Medical University, Asahikawa, Hokkaido, Japan

**Keywords:** foot infection, elderly patient, chronic limb-threatening ischemia

## Abstract

Chronic limb-threatening ischemia (CLTI) is an important issue for elderly patients with peripheral artery disease. Here, we present the case of a 91-year-old man with CLTI, residing in a rural district. The onset of CLTI rapidly deprived him of ambulation because of a foot infection. Given that he had difficulty with long-distance transportation, limb salvage for extensive tissue loss was performed at a district facility, based on his and his family’s request. Finally, skin grafting on the cutting plane of the right ankle bones resulted in wound healing in six months after incomplete revascularization and multiple minor amputations.

## Introduction

Peripheral artery disease (PAD) is a clinical issue for the elderly because arteriosclerotic risk factors such as diabetes mellitus (DM) and renal disorders contribute to developing arterial occlusive lesions in an aging society. Chronic limb-threatening ischemia (CLTI) is a clinical syndrome associated with mortality, amputation, and impaired quality of life (QOL) due to PAD.^1)^ For elderly people, major amputation and impaired QOL accelerate loss of ambulation and malnutrition-limited indications of lower extremity revascularization.^2–4)^ To address the difficulty of developing a treatment strategy for CLTI in elderly people, we report a case that achieved wound healing on the cutting plane of the ankle bones with incomplete revascularization (IR) for extensive tissue loss, with deep infection.

## Case Report

**Figure 1:**
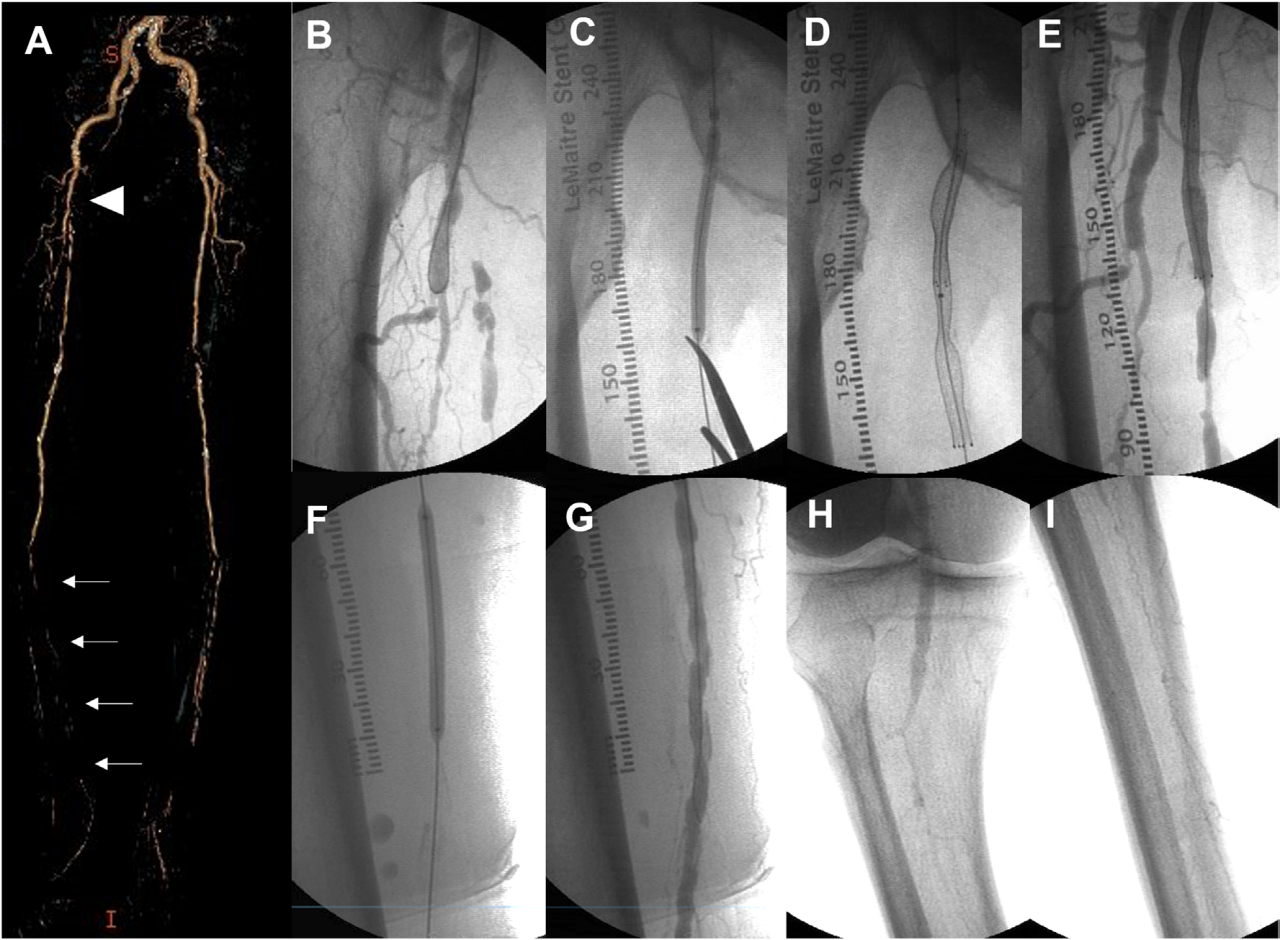
Fig. 1 Findings of revascularization. Preoperative computed tomography angiography showing diseased right superficial femoral artery (SFA, arrowhead) and chronic total occlusion (CTO) of the infrapopliteal arteries (arrows) (**A**). Additionally, the angiography demonstrates CTO of a proximal segment of the right SFA (**B**). A guidewire was crossed into the CTO lesions by endarterectomy of the distal SFA, and stents were placed after balloon angioplasty was performed (**C**, **D**). A distal segment of the right SFA was stenosed (**E**), and balloon angioplasty was performed (**F**, **G**). Infrapopliteal arteries were diseased; however, a contrast equipment was poor to show these segments (**H**, **I**).

Here, we present a 91-year-old man, residing in a rural district, with hypertension and DM. He visited a university hospital to consult for a formation of an ulcer on his right second toe one year ago, where CLTI was diagnosed. His ankle brachial pressure index (ABI) was 0.62, and skin perfusion pressure (SPP) showed 24 mmHg on the dorsum and 27 mmHg on the plantar surface. His right femoral artery was palpable; however, the popliteal artery and foot arteries were not. Femoropopliteal and infrapopliteal arterial lesions were indicated based on the physical examination. Despite his ambulatory status, revascularization was not indicated due to his age and the small size of the ulcer, and observation was recommended. Unfortunately, the condition of his right second toe worsened, progressing to infectious gangrene, resulting in severe foot pain and difficulty in ambulation. His white blood cell count was 8,700/mm^3^, and his C-reactive protein level was 8.02 mg/dL. Primary amputation was provided from the clinic to the patient and his family; however, he and his family requested limb salvage. Additionally, because of the patient’s old age and his family’s wishes, the patient and his family hoped that the treatment could be performed in the district clinic without having to transfer to a limb salvage center because of their difficulties in commuting. The clinical issues addressed for CLTI treatment and limb salvage at the clinic were as follows: (1) digital subtraction angiography (DSA) equipment was available but insufficient to assess the entire segment of the lower extremity, as well as difficulty in image magnification and reduction; (2) general anesthesia was not available for this high-risk patient; and (3) endovascular therapy (EVT) was possible for the iliac and femoral segment using the DSA equipment, but surgical repair was difficult when a complication occurred after EVT. The common femoral artery was palpable, and computed tomography angiography showed no iliac artery lesion but chronic total occlusion (CTO) of the right superficial femoral artery (SFA) (**Fig. 1A**). Only the right SFA was revascularized under local anesthesia; partial endarterectomy of the severe calcification of the SFA was performed to cross a guidewire into the CTO lesion, and 7.0×150-mm SMART (Cardinal Health Japan G.K., Tokyo, Japan) was placed for the arterial lesion (**Figs. 1B**–**1G**). Infrapopliteal arteries were not assessed well because of the equipment ability and patient’s movement (**Figs. 1H**–**1I**). The right popliteal artery was palpable after the revascularization. Doppler sound of the right posterior tibial artery was also improved. The ABI increased to 0.82 postoperatively. SPP was not evaluated after EVT, given that it was unavailable in the clinic.

**Figure 2:**
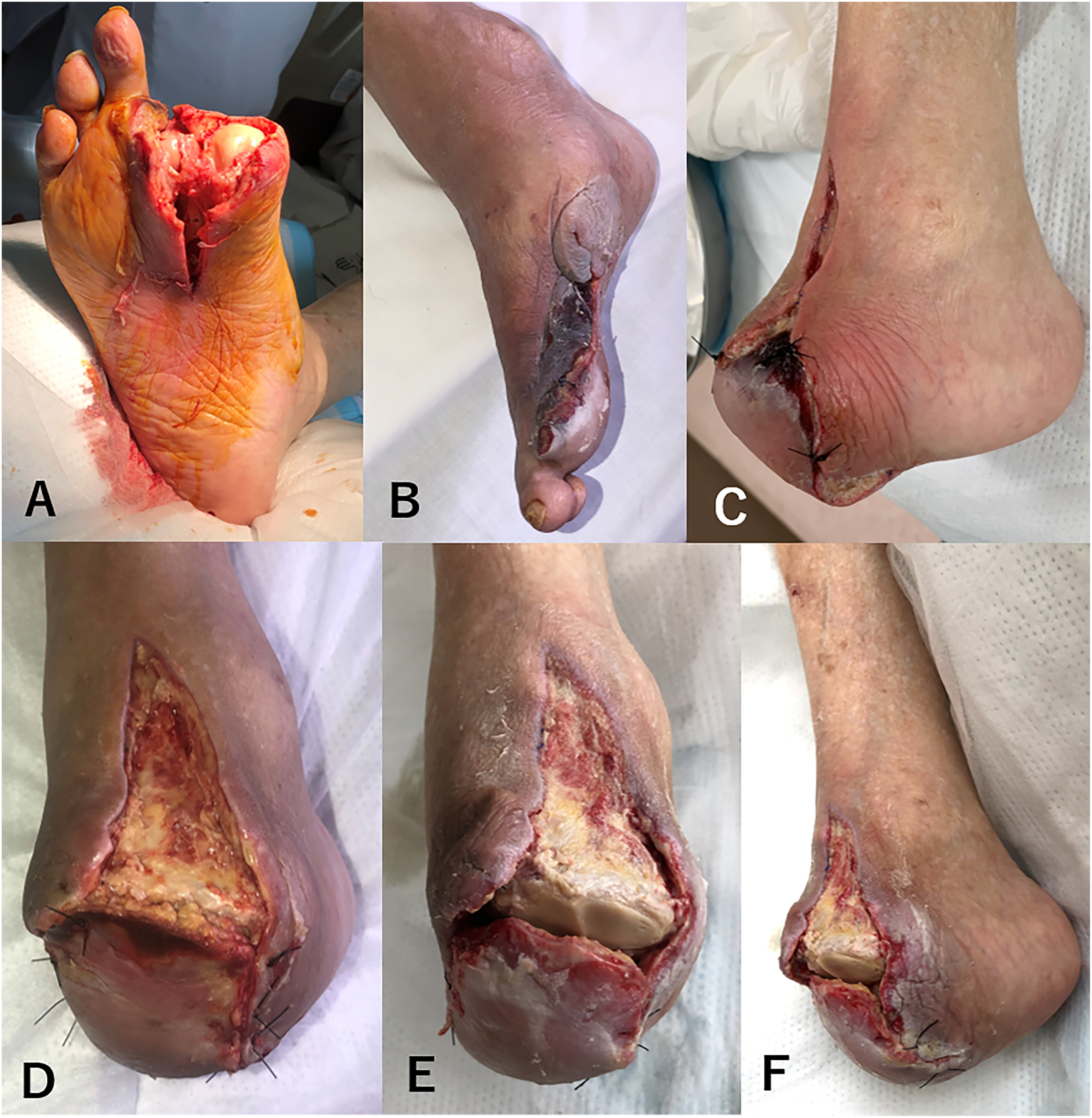
Fig. 2 Images of infectious gangrene of the right foot. Minor amputation of the first and second toe and incision and drainage of plantar were performed (**A**). Next, the first to third metatarsal bones were resected. The residual skin was closed, but the wound was subsequently infected (**B**). Lisfranc amputation was performed; however, wound infection was not controlled (**C**), the Chopart amputation was finally performed, and the residual skin was closed (**D**). However, the infection was still active, and ankle bones were exposed (**E**, **F**).

**Figure 3:**
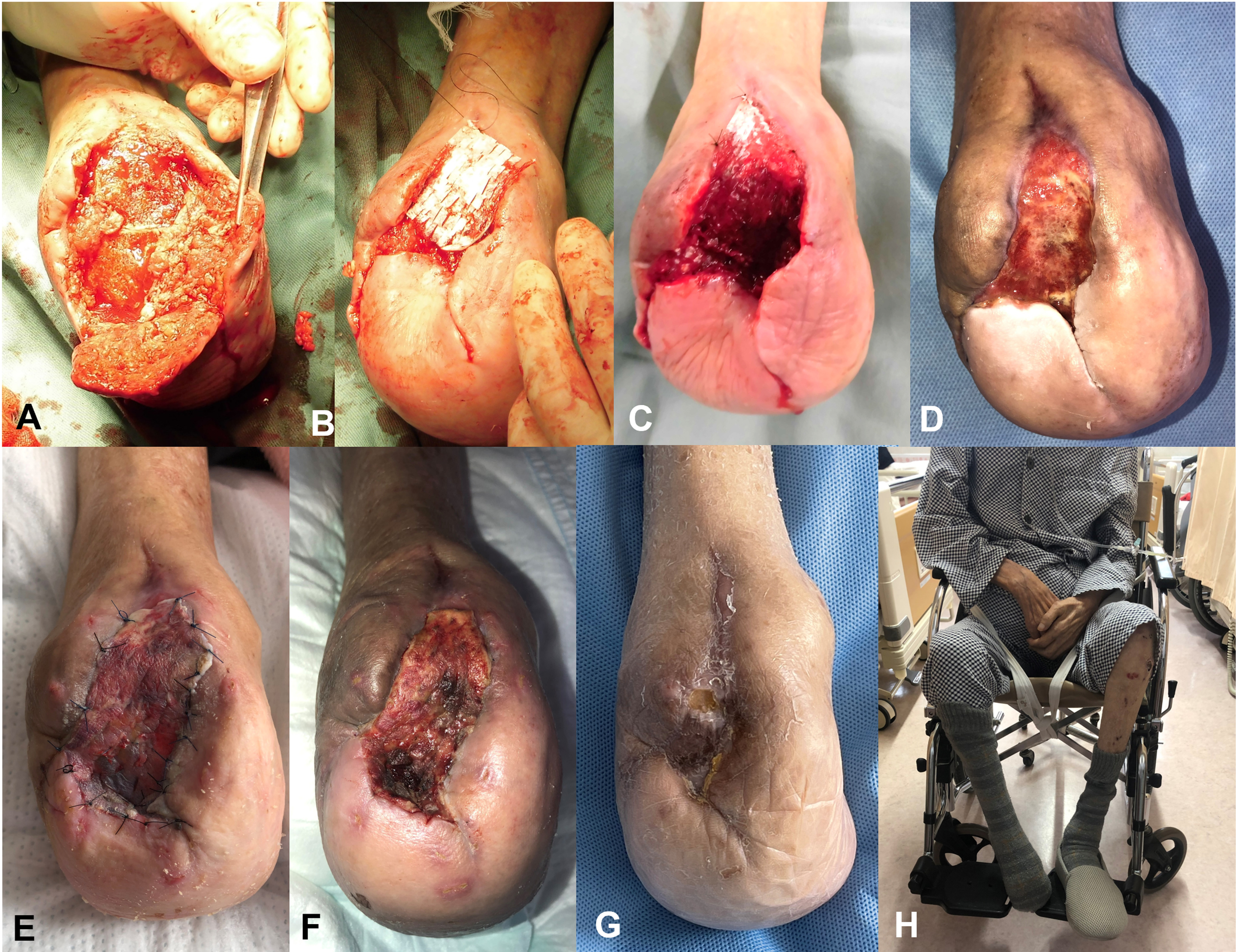
Fig. 3 Process of wound healing. Infectious tissues were resected, and exposed bones were partially shaved (**A**). After suturing the residual skin, an artificial dermis was placed on the bone marrow (**B**) 3 days (**C**) and 21 days (**D**) after the artificial dermal placement. Skin grafting was performed 50 days after the artificial dermal placement (**E**). Skin grafting attached well 7 days after grafting (**F**). Wound healing was accomplished 143 days after skin grafting (**G**). The right limb was salvaged and made functional for transfer to a wheelchair (**H**).

The infected second toe was amputated, and the condition of the first toe also worsened. The infection still spread into the plantar area. Minor amputation of the first toe was performed, and surgical incision and wound drainage were added (**Fig. 2A**). In the wound culture, methicillin-resistant *Staphylococcus aureus* was detected, and vancomycin was administered. Multiple minor amputations, including resection of metatarsal bones and Lisfranc amputation, were performed. However, the infection was not well controlled (**Figs. 2B** and **2C**). Chopart amputation was finally required, and additional incisional drainage was performed on the infected areas of the dorsum (**Figs. 2D**–**2F**). Granulation grew gradually, but not around the talus bone. We cut the talus and heel bones’ surfaces and exposed the bone marrow on the 80th hospitalized day to epithelialize this wound. An artificial dermis, OASIS collagen matrix (Cook Medical Corporation G.K., Tokyo, Japan), was covered to develop scaffolds of granulation, given that bleeding from the marrow was well observed (**Figs. 3A**–**3C**). The development of granulation was smooth (**Fig. 3D**). On the 123rd hospitalized day, skin grafting was performed. A full-thickness skin graft was harvested from the right thigh under local anesthesia, and it was transplanted onto the defected area. The harvested site was directly sutured (**Fig. 3E**), and wound healing progressed gradually (**Fig. 3F**). Finally, the wound completely healed on the 173rd hospitalized day (**Fig. 3G**). He could operate a wheelchair independently, and the salvaged right limb helped him get on and off the wheelchair (**Fig. 3H**). Unfortunately, he died three months after wound healing due to pulmonary aspiration.

## Discussion

In an aging society, the incidence of PAD is increasing in elderly people. The metabolic syndrome is recognized as a risk factor for PAD development, the prevalence of which increases with age,^5)^ especially the prevalence of DM is strongly correlated with aging. Older age is a potential predictor of major amputation and mortality in patients with diabetes and foot ulcers.^6)^ They usually have more peripheral neuropathy and poly-vascular diseases. As shown in the current case, the prognosis of patients in the 90s cannot be expected, leading to primary amputation, and EVT might be indicated as a treatment for CLTI in high-risk patients.

The number of elderly people with PAD is expected to increase in rural areas. Limb salvage centers are still not enough in Japan, especially in Hokkaido, increasing major amputations and performance of inappropriate treatment. As shown here, DSA equipment for angiography and angioplasty and adequate medical knowledge are underserved because there is no stand-by vascular surgeon on-site. Especially, the management of complications after EVT should be considered; EVT was safely performed in a rural faculty. However, 10% of complications occurred, of which 0.66% were classified as major, such as wire retention and retroperitoneal hemorrhage.^7)^ High technical success and a low complication rate are desirable, given that vascular-related complications are associated with a patient’s prognosis.

Moreover, frail patients who undergo high-stress vascular procedures have a significantly higher rate of complications, leading to loss of function and independence and mortality after surgery.^8)^ In the current case, we limited revascularization for PAD by the femoral artery lesions because technical success was not guaranteed in infrapopliteal artery diseases under insufficient resources. For CLTI patients, complete revascularization, which achieves inline flow to the foot through at least one patent tibioperoneal artery, is desirable. Meanwhile, IR increases the risk of major amputation.^9)^ In the current case, IR or residual infrapopliteal artery disease may cause difficulty in infection control and the need for multiple bone resections. Finally, the infection was kept under control by Chopart amputation. However, the foot tissue was extensively lost.

There are skin flaps, free flaps, and skin grafting to heal the extensive tissue loss. The skin flap was difficult to apply because the residual skin was infected and resected (**Fig. 2E**). The free flap was also inappropriate because there was no reliable inflow artery around the wound, and the surgical intervention was too invasive for the patient. The only option for soft tissue defected with exposed bones in the clinic was skin grafting. The exposed bone was shaved, and the bone marrow was exposed. Blood flow was confirmed from the bone marrow, and an artificial dermis was placed on the bone marrow to increase granulation. Specific bone marrow cells can transform into epithelial cells and assist the recipient’s uptake of skin grafts.^10)^ Although skin grafting on the bone marrow may be theoretically reasonable, long-term durability is unclear because of the exacerbation of infections such as osteomyelitis. Although major amputation was considered before a series of operations, the salvaged limb was functional. The patient and his family were satisfied with our surgical procedures. In terms of medical cost, limb salvage for this patient was not justified because the surgery would have cost 240,000 yen for primary amputation, compared with about 1,200,000 yen for a series of treatments for CLTI. The cost would have been five times higher had the limb been salvaged. To reduce the medical costs, a curable line of damaged foot should be expected, and short treatment time with infrequent surgical procedures is essential if limb salvage is opted for elderly patients.

## Conclusion

Limb salvage is usually difficult for elderly people with CLTI, especially in rural district facilities. Although insufficient resources lead to limited treatment, wound healing contrivance may achieve limb salvage. Therefore, we reported a case of wound healing by skin grafting on the cutting plane of ankle bones after infectious gangrene due to CLTI.

## References

[1] Conte MS, Bradbury AW, Kolh P, et al. Global vascular guidelines on the management of chronic limb-threatening ischemia. Eur J Vasc Endovasc Surg 2019; 58: S1-S109.e33. 31182334 10.1016/j.ejvs.2019.05.006 PMC8369495

[2] Peters CML, de Vries J, Lodder P, et al. Quality of life and not health status improves after major amputation in the elderly critical limb ischaemia patient. Eur J Vasc Endovasc Surg 2019; 57: 547-53. 30826247 10.1016/j.ejvs.2018.10.024

[3] Flu HC, Lardenoye JH, Veen EJ, et al. Functional status as a prognostic factor for primary revascularization for critical limb ischemia. J Vasc Surg 2010; 51: 360-71.e1. 20141960 10.1016/j.jvs.2009.08.051

[4] Matsubara Y, Matsumoto T, Aoyagi Y, et al. Sarcopenia is a prognostic factor for overall survival in patients with critical limb ischemia. J Vasc Surg 2015; 61: 945-50. 25498160 10.1016/j.jvs.2014.10.094

[5] Kohara K. Sarcopenic obesity in aging population: current status and future directions for research. Endocrine 2014; 45: 15-25. 23821364 10.1007/s12020-013-9992-0

[6] Costa RHR, Cardoso NA, Procópio RJ, et al. Diabetic foot ulcer carries high amputation and mortality rates, particularly in the presence of advanced age, peripheral artery disease and anemia. Diabetes Metab Syndr 2017; 11 **Suppl 2**: S583-7. 28465149 10.1016/j.dsx.2017.04.008

[7] Ansari A, Shah MA, Shah MA, et al. Safety of day-case endovascular interventions for peripheral arterial disease in a rural, underserved area. Ther Adv Cardiovasc Dis 2020; 14: 1753944720948651. 32885724 10.1177/1753944720948651 PMC7475785

[8] Kikuchi S, Sasajima T, Inaba M, et al. Evaluation of paramalleolar and inframalleolar bypasses in dialysis- and nondialysis-dependent patients with critical limb ischemia. J Vasc Surg 2018; 67: 826-37. 28965798 10.1016/j.jvs.2017.07.116

[9] Takayama T, Matsumura JS. Complete lower extremity revascularization via a hybrid procedure for patients with critical limb ischemia. Vasc Endovascular Surg 2018; 52: 255-61. 29486676 10.1177/1538574418761723

[10] Tamai K, Yamazaki T, Chino T, et al. PDGFR alpha-positive cells in bone marrow are mobilized by high mobility group box 1 (HMGB1) to regenerate injured epithelia. Proc Natl Acad Sci USA 2011; 108: 6609-14. 21464317 10.1073/pnas.1016753108 PMC3081004

